# Gut microbiota–host immunity crosstalk in psoriasis: from dysbiosis to cutaneous inflammation

**DOI:** 10.3389/fimmu.2026.1957850

**Published:** 2026-10-09

**Authors:** Meng Zhang, Shengni Zhang, Xiangxiang Ren

**Affiliations:** 1 Department of Dermatology, Affiliated Hospital of Hebei University, Baoding, China; 2 Department of General Surgery, Affiliated Hospital of Hebei University, Baoding, China

**Keywords:** gut microbiota, gut–skin axis, IL-23/IL-17 axis, intestinal barrier, psoriasis, short-chain fatty acids

## Abstract

Psoriasis is a chronic inflammatory skin disease driven by the IL-23/IL-17 axis. Evidence accumulated over the past decade indicates that its pathogenesis extends beyond the skin and involves bidirectional communication between the intestinal microbiota and host immunity. This narrative review organizes current evidence along a proposed sequence running from gut dysbiosis and intestinal barrier damage, through altered microbial metabolites, to reprogrammed host immune responses and cutaneous inflammation. We summarize the gut microbiota changes reported in patients with psoriasis and the clinical evidence for impaired intestinal permeability. We describe how microbiota-derived metabolites—short-chain fatty acids (SCFAs), tryptophan/kynurenine metabolites, and bile acids—modulate Th17/Treg balance and innate immunity through the aryl hydrocarbon receptor (AhR) and G protein-coupled receptors. We then review clinical data on microbiome-directed interventions, chiefly probiotics and fecal microbiota transplantation (FMT), and close with the outstanding issues in causal inference, methodological heterogeneity, and standardization of interventions. The current evidence is consistent with the gut–skin axis hypothesis. The direction of causality between the microbiota and psoriasis, the dose-response relationship, and the clinical benefit of microbiome-directed therapy nonetheless remain to be confirmed in prospective cohorts and well-designed randomized controlled trials.

## 1 Introduction

Psoriasis affects roughly 1%–3% of the global population. Its core pathology is abnormal activation of cutaneous innate and adaptive immunity, which culminates in IL-23/IL-17-driven keratinocyte hyperproliferation and an inflammatory microenvironment (1, 2). Over the past decade, attention has moved beyond the skin-resident microbiome to the intestine, the body’s largest microbial reservoir, and its functional connection to the skin—a link now commonly called the gut–skin axis (3–5).

Evidence for the gut–skin axis rests on three lines of observation. First, the composition of the gut microbiota in patients with psoriasis differs from that of healthy controls, and some of these differences track with disease severity (6, 7). Second, animal studies show that the intestinal microbiota can shape cutaneous inflammation through immune pathways; germ-free conditions or antibiotic treatment attenuate imiquimod (IMQ)-induced psoriasiform dermatitis (8, 9). Third, microbiome-directed interventions such as probiotics and FMT have improved clinical outcomes in some studies (10, 11). Much of this evidence, however, comes from cross-sectional observations or extrapolation from animal models, and the direction of causality, the responsible taxa, and the metabolic mediators remain unsettled (12).

This review follows the axis of dysbiosis, barrier/metabolite changes, immune responses, and skin inflammation. It does not attempt to cover every reported association between the microbiota and psoriasis; comorbidities and drug responses are mentioned only where directly relevant. The goal is to synthesize the evidence for the mechanistic model and to identify the gaps that future studies should address.

## 2 Clinical associations between the gut microbiota and psoriasis

### 2.1 Findings from case-control and cohort studies

Several case-control studies have compared fecal microbiota between patients with psoriasis and healthy controls. Early 16S rRNA-based studies reported altered gut microbial diversity and changes in the Firmicutes/Bacteroidetes ratio, although the results were not fully consistent across cohorts (6, 7). A Brazilian case-control study that matched for age, sex, and body mass index still observed differences in the microbial profile, arguing that the changes cannot be explained by obesity alone (13). Metagenomic analyses have gone a step further by identifying putatively beneficial species, such as Blautia wexlerae, alongside enrichment of potential pathobionts (7).

### 2.2 Associations with disease severity

Evidence linking microbial composition to disease severity comes mainly from cross-sectional studies. Cytokine profiles correlate with the alpha and beta diversity of the gut microbiota in psoriasis (6), and patients with concurrent metabolic syndrome show a microbial signature distinct from that of patients with psoriasis alone (14). Longitudinal clues come from intervention studies: after treatment with secukinumab (15) or IL-17A inhibitors (16), and after biologic therapy more generally (17), the intestinal and cutaneous microbiota change, suggesting that microbial status tracks disease activity rather than remaining fixed.

### 2.3 Inconsistency and confounding

The robustness of the microbiota–psoriasis association remains debated. A matched-pair deep-sequencing study detected only subtle shifts in the microbiome, which were associated with elevated fecal calprotectin rather than with broad compositional change (12). Another study found no significant change in serum or fecal calprotectin in patients with psoriasis (18). Sample size, sequencing platform, ethnicity, and diet may all contribute to discrepancies among studies. In addition, most case-control reports did not fully account for recent antipsoriatic treatment, which can itself alter the gut microbiota (17); larger prospective designs that record therapy history are needed to define a reproducible microbial signature. The direction and consistency of the evidence across these findings are summarized in **Table 1**.

**Table 1:** Summary of evidence direction and consistency across major findings in psoriasis and gut microbiota research.

Finding area	Reported direction	Consistency	Refs
Gut alpha diversity	Reduced	Mixed	(6, 12, 14)
Firmicutes/Bacteroidetes ratio and SCFA producers	F/B variable; SCFA producers ↓	Mixed	(6, 7)
Akkermansia muciniphila (IMQ model)	Protective	Consistent (animal)	(19)
Prevotella copri	Aggravating	Emerging	(20)
Claudin-3/I-FABP	Elevated	Consistent	(24–27)
Intestinal permeability (LPS, TMAO)	Increased	Consistent	(22, 23, 26)
SCFAs	Anti-inflammatory	Consistent	(8, 30–33)
Tryptophan/I3S/ILA	Dual roles	Emerging	(9, 34, 35, 39)
Kynurenine pathway	Dysregulated	Consistent	(36, 37)
Bile acids	Altered	Emerging	(21, 38)
Th17/Treg balance	Th17 ↑, Treg ↓	Consistent	(1, 2, 40)
Probiotics (RCTs)	Mild benefit	Consistent but underpowered	(10, 45–47)
Fecal microbiota transplantation	Anecdotal	Insufficient	(11, 48, 49)

F/B, Firmicutes/Bacteroidetes; IMQ, imiquimod; SCFA, short-chain fatty acid; I3S, indoxyl sulfate; ILA, indole-3-lactic acid; LPS, lipopolysaccharide; TMAO, trimethylamine N-oxide; RCT, randomized controlled trial. Consistency categories were assigned from the studies listed in the Refs column: Consistent, the direction was reproduced in multiple independent studies and not contradicted within the same row; Mixed, the cited studies reported conflicting directions (for example, the Firmicutes/Bacteroidetes ratio is classified as Mixed because the studies cited in Section 2.1 were not fully concordant across cohorts); Emerging, the evidence is limited but concordant; Insufficient, evidence is anecdotal or not yet tested in adequately powered trials. The Refs column lists all studies on which each row’s assessment is based; rows with fewer than three cited studies should be read as provisional.

↑, increase; ↓, decrease.

## 3 Features of gut dysbiosis in psoriasis

### 3.1 Reduced diversity and altered community structure

Most studies report decreased alpha diversity or a beta diversity shift away from the healthy profile in patients with psoriasis, although the direction of change varies among cohorts (6, 12, 14). Commonly described compositional alterations include an increase in the relative abundance of opportunistic pathogens and a reduction in SCFA-producing taxa such as Faecalibacterium and Roseburia (7).

### 3.2 Taxa of interest

Functional studies provide mechanistic clues at the genus level. Akkermansia muciniphila alleviates skin thickening and intestinal inflammation and partially restores the microbiota in the IMQ model (19). Prevotella copri, by contrast, aggravates psoriasiform inflammation through keratinocyte-neutrophil crosstalk involving NF-κB (20). Work framed within traditional Chinese medicine has characterized the dampness-heat pattern of psoriasis by reduced Lactobacillus abundance and accumulation of deoxycholic acid (21). Together, these observations indicate that genus-level effects are both strain-specific and direction-specific.

## 4 Host-microbiota immune interactions

### 4.1 Impaired intestinal barrier

The intestinal barrier consists of the epithelium, tight junctions, and mucosal immunity. Loss of its integrity, commonly described as a leaky gut, permits bacterial components such as LPS and microbial metabolites to enter the circulation and is regarded as a key initiating step of the gut–skin axis (22, 23). In clinical studies, serum claudin-3 and intestinal fatty acid-binding protein (I-FABP) are elevated in patients with plaque psoriasis (24–27), with some studies reporting an association with disease severity (26, 27). Patients with mild-to-moderate disease also show subclinical duodenal inflammation and a tendency toward barrier disturbance (23). In animal models, IMQ-induced skin inflammation is accompanied by intestinal inflammation and barrier dysfunction (28). At the molecular level, microRNA-31-3p compromises the skin barrier by targeting CLDN8, pointing to systemic regulation of barrier integrity (29). Circulating trimethylamine N-oxide (TMAO), a gut microbiota-derived metabolite, provides further evidence of microbial translocation in psoriasis. Patients with an impaired intestinal barrier have higher plasma TMAO than those with intact barrier function (26), supporting its use as a circulating marker of barrier compromise.

### 4.2 Microbial metabolites: SCFAs, tryptophan derivatives, and bile acids

SCFAs. Acetate, propionate, and butyrate are the main products of microbial fermentation of dietary fiber and regulate immunity through the G protein-coupled receptors GPR43/41 and through histone deacetylase inhibition (2, 8). In the IMQ model, SCFA supplementation reduces skin thickening and IL-17 levels while altering gut microbial composition (8). The butyrate receptor HCAR2/GPR109A controls IMQ-induced skin inflammation (30), and crosstalk among the microbiota, regulatory T cells (Tregs), and HCA2 has been characterized in psoriatic models (31). Clinical and review-level evidence supports a role for SCFAs in modulating Th17 differentiation and Treg function (2, 32, 33).

Tryptophan metabolism. Tryptophan is converted through the indole pathway into derivatives that act as endogenous ligands of AhR. Indole-3-lactic acid (ILA) suppresses IL-17A in an AhR-dependent manner and ameliorates IMQ-induced dermatitis (34), and inhibits keratinocyte proliferation (35). A 2025 study in Immunity demonstrated that indole-producing intestinal microbes promote host synthesis of indoxyl sulfate (I3S) through a metabolic relay; I3S then signals through AhR in cutaneous Th17 cells, modulating chromatin accessibility and amplifying skin inflammation (9). This work directly demonstrates that gut microbial metabolites can reprogram cutaneous immunity at a distance. Multiple studies have linked dysregulation of the kynurenine pathway to psoriatic inflammation and identified it as a candidate therapeutic target (36, 37).

Bile acids. As host metabolites modified by the microbiota, bile acids have recently entered the gut–skin axis framework. Microbiota-induced perturbation of bile acids alters keratinocyte lipid metabolism (38), and the dampness-heat pattern of psoriasis combines deoxycholic acid accumulation with reduced Lactobacillus abundance (21). Quinolinic acid, a tryptophan metabolite produced by the skin microbiota, negatively regulates the NLRP3 inflammasome through AhR, illustrating that metabolites can exert bidirectional control over inflammation (39).

These metabolites are not interpreted passively by the skin. Keratinocytes express AhR and respond to tryptophan derivatives such as indole-3-lactic acid and indoxyl sulfate, adjusting their production of antimicrobial peptides and chemokines (34, 35). Gut microbe-derived metabolites also act on skin Th17 cells (9); SCFAs act on keratinocytes and skin-resident dendritic cells to restrain local inflammation (30, 31), and bile-acid receptors are present on several cutaneous immune populations (38). The skin therefore reads gut-derived signals through its own receptor repertoire and feeds back through keratinocyte cytokines; the axis is a two-way conversation rather than a one-way pipe.

### 4.3 Immune pathways

The IL-23/IL-17 axis is central to the immunopathology of psoriasis, and the microbiota and its metabolites modulate it through several routes. SCFAs suppress Th17 polarization and IL-17 production (2, 8); the I3S–AhR pathway enhances the pathogenicity of cutaneous Th17 cells (9); IL-17A inhibitor therapy is accompanied by changes in the microbiota (16); and treatment targeting the tyrosine kinase 2 (TYK2)/IL-23 pathway is associated with changes in IL-6 and tryptophan metabolites (1). Treating IL-23 and IL-17 as a single co-directional unit, however, obscures a tissue asymmetry that the gut–skin axis makes explicit.

Treg/Th17 imbalance represents a second core mechanism. Patients with psoriasis have functional defects in Tregs (40). Microbiota-derived SCFAs acting through HCAR2 and HCA2 promote Treg expansion and suppress Th17 polarization, thereby regulating cutaneous inflammation (30, 31). This framework applies to the skin and to circulating pathogenic Th17 cells; in the intestinal mucosa, Th17 cells are normal residents that maintain barrier function and are not inherently pathogenic. The two compartments must therefore be distinguished when interpreting how microbial signals shape Th17 behavior. Positive feedback loops involving the microbiota, immune cells, and keratinocytes, such as Prevotella copri-driven NF-κB crosstalk and direct action of microbial metabolites on keratinocytes, amplify and sustain local inflammation (20, 35, 38).

The clinical importance of this distinction becomes clear when IL-23 and IL-17 are separated. IL-23 is pathogenic in both inflammatory bowel disease and psoriasis, which is why the selective IL-23 p19 inhibitors (risankizumab, guselkumab) and the IL-12/23 p40 inhibitor ustekinumab are effective, and safe, in both conditions (41). IL-17, by contrast, has context-dependent effects in the intestine, where IL-17A supports barrier defense in part by inducing antimicrobial peptides (42). This is why the clinical consequences of IL-17 blockade differ by drug and indication: brodalumab is formally contraindicated in Crohn’s disease, whereas secukinumab and ixekizumab carry warnings for inflammatory bowel disease and require caution and monitoring (43). The gut–skin sequence is therefore tissue-asymmetric: gut dysbiosis that raises IL-23 production by intestinal dendritic cells produces opposite IL-17 outcomes in the gut (barrier protection) and the skin (keratinocyte hyperproliferation and inflammation). This asymmetry also bears on the microbiome changes seen during biologic therapy. Because IL-17A inhibitors alter the gut microbiota (15, 16), it is an open and clinically relevant question whether those changes contribute to the risk of colitis, and whether IL-23 inhibitors, which spare gut-protective IL-17, leave a different, potentially safer microbial signature (17). Microbiome-directed adjuncts that restore SCFA-producing taxa and Treg function might therefore be especially useful for patients on IL-17 inhibitors, a possibility that deserves prospective testing.

### 4.4 A conceptual model of the gut–skin axis

The evidence above can be integrated into a sequence with defined transitions. (i) Gut dysbiosis—loss of diversity, depletion of SCFA producers, enrichment of pathobionts—is documented in case-control and metagenomic studies (6, 7, 12). (ii) Barrier damage, reflected by elevated claudin-3, I-FABP and LPS and by tight-junction disruption, follows in several independent cohorts (22, 24–27). (iii) The altered metabolite landscape—reduced SCFAs and abnormal indole/I3S and bile-acid profiles—has been characterized functionally in animal models and in emerging human data (8, 9, 21, 30, 31, 38). (iv) Host immune reprogramming then diverges by tissue: in the skin, IL-23 and IL-17 drive keratinocyte hyperproliferation, whereas in the gut IL-17 maintains barrier integrity, and the balance between these outputs, together with Treg/Th17 imbalance and innate activation, determines net inflammation. (v) Skin inflammation is the final outcome. Signals also travel in reverse: cutaneous UVB exposure alters the gut microbiota independently of skin inflammation in mice (44), although clinical confirmation is lacking. The weakest link in this chain is the transition from metabolite changes to tissue-specific immune reprogramming, because most supporting evidence is correlative or derived from animals; the more robust steps are the association between dysbiosis and psoriasis and the clinical evidence for barrier damage. The model should therefore be read as a working hypothesis. Three caveats deserve emphasis. First, reverse causation has not been excluded: chronic cutaneous inflammation may alter circulating mediators that in turn affect intestinal function and microbial composition. Second, shared upstream drivers—diet, medication, and psychological stress—can influence both the gut microbiota and psoriasis independently, and their contribution has rarely been quantified. Third, skin-to-gut signaling in humans remains unconfirmed. Each step requires validation in prospective cohorts with repeated sampling. The model is falsifiable: if prospective data showed metabolic recovery without compositional restoration of the microbiota, or barrier restoration preceding microbial change, the proposed ordering would need revision. A schematic of the pathway, with evidence strength distinguished by the nature of the supporting studies, is shown in **Figure 1**.

**Figure 1:**
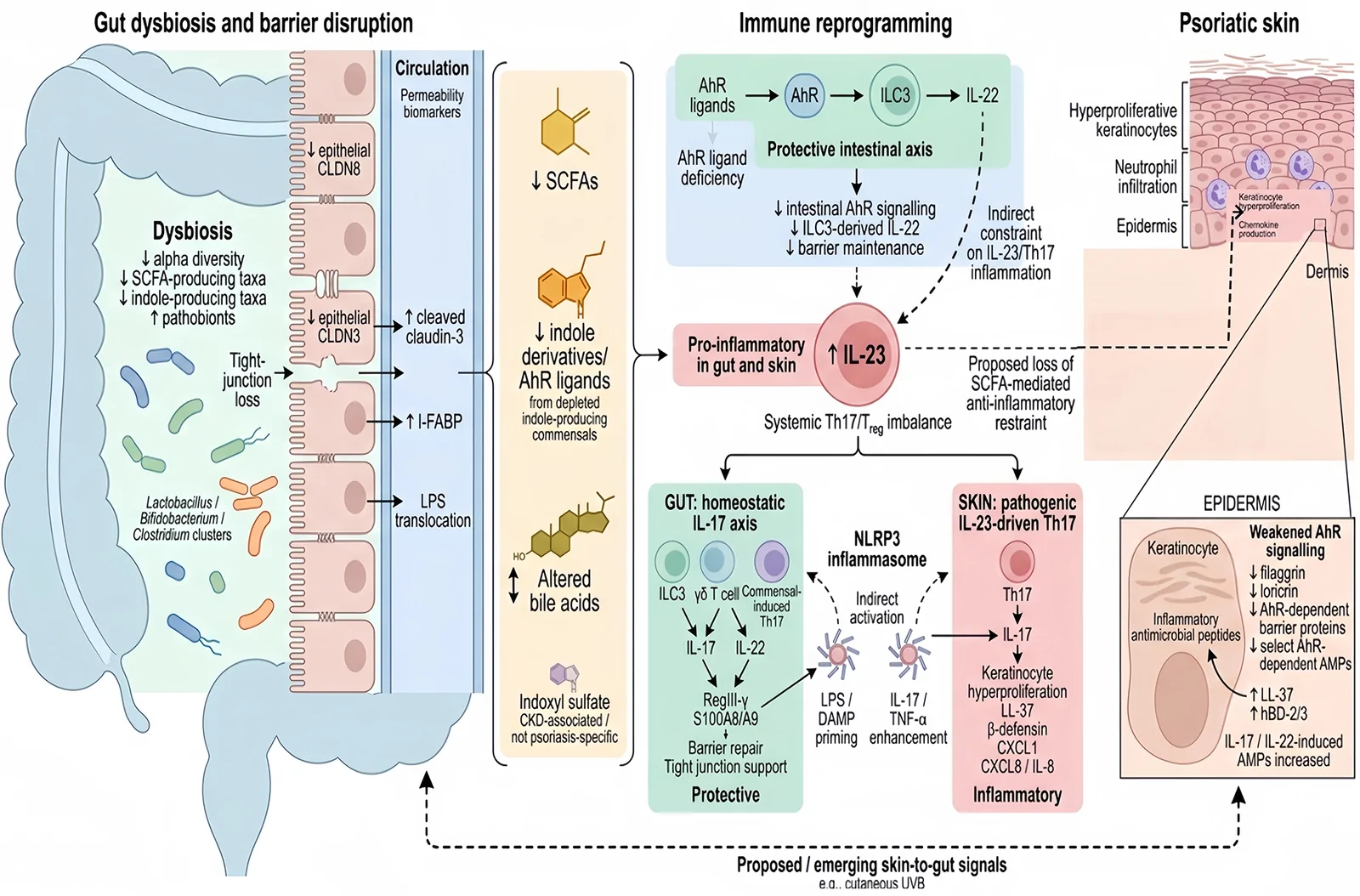
Gut microbiota–host immunity interactions in psoriasis: a mechanistic model. The proposed pathway runs from gut dysbiosis (reduced alpha diversity, depletion of SCFA-producing and indole-producing taxa, enrichment of pathobionts) through intestinal barrier damage (reduced epithelial claudin-3 and claudin-8; elevated circulating cleaved claudin-3 and intestinal fatty acid-binding protein (I-FABP); lipopolysaccharide (LPS) translocation), altered microbial metabolites (reduced SCFAs and reduced indole derivatives/AhR ligands; altered bile acids), host immune reprogramming (AhR ligand deficiency impairing intestinal homeostasis through reduced ILC3-derived IL-22 and weakened barrier maintenance; IL-23 upregulation; tissue-divergent IL-17 outcomes—gut-protective IL-17 and IL-22 from homeostatic sources versus IL-23-driven pathogenic IL-17 in skin; systemic Th17/Treg imbalance; indirect NLRP3 inflammasome activation in skin and gut), and finally cutaneous inflammation (keratinocyte hyperproliferation, IL-17-induced antimicrobial peptide (LL-37/β-defensin) and chemokine (CXCL1/CXCL8) production, neutrophil infiltration, reduced AhR-dependent barrier proteins such as filaggrin/loricrin, and loss of SCFA-mediated anti-inflammatory restraint, shown as emerging/proposed). Bidirectional skin-to-gut signals (e.g., proposed cutaneous UVB exposure) are indicated. AhR, aryl hydrocarbon receptor; I-FABP, intestinal fatty acid-binding protein; IL, interleukin; ILC3, group 3 innate lymphoid cell; LPS, lipopolysaccharide; NLRP3, NOD-, LRR- and pyrin domain-containing protein 3; SCFA, short-chain fatty acid; Th, T helper; Treg, regulatory T cell; UVB, ultraviolet B.

## 5 Therapeutic targets

The interventions discussed in this section act on distinct steps of the pathway outlined in **Figure 2**.

**Figure 2:**
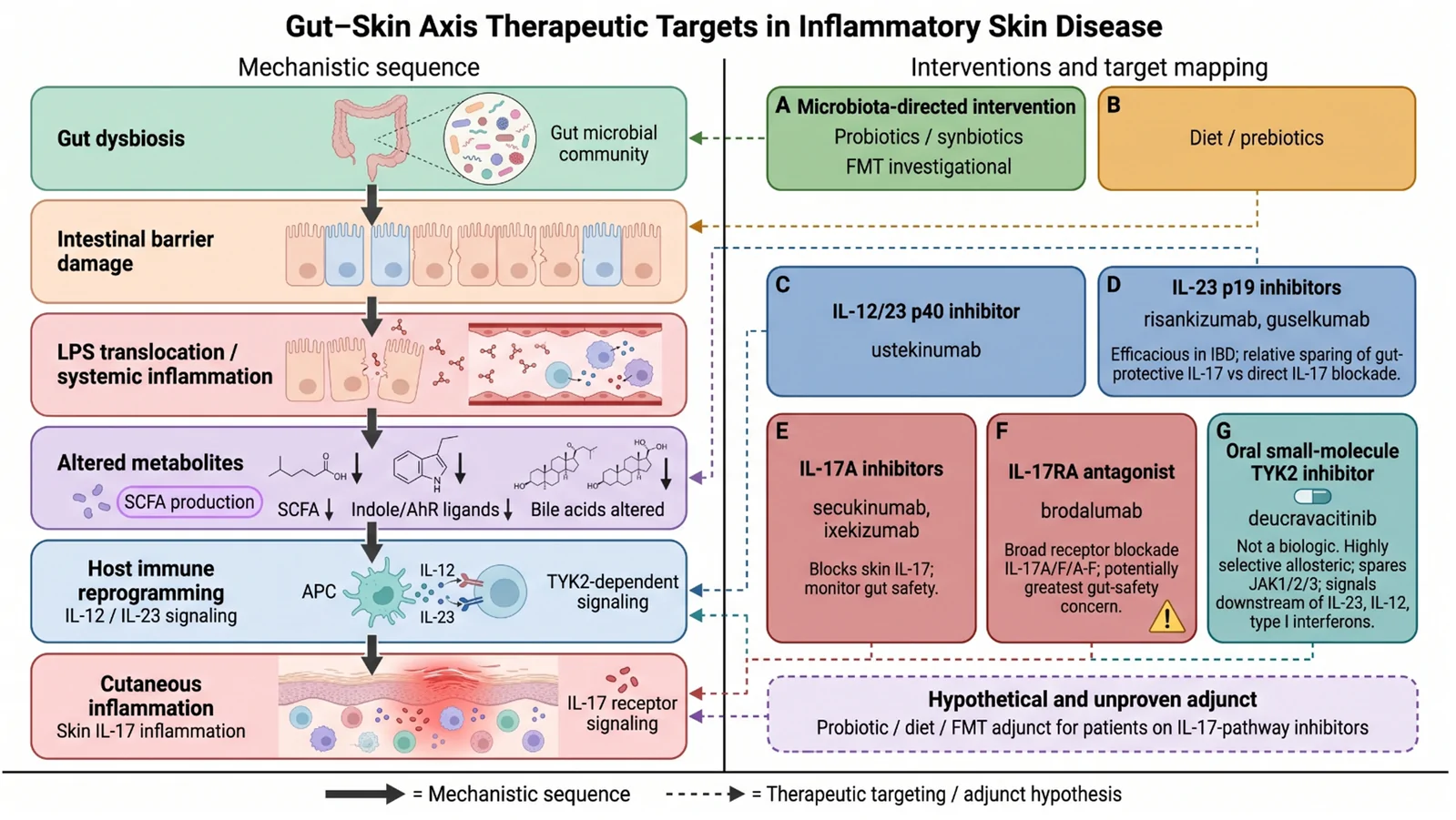
Therapeutic interventions along the gut–skin axis in psoriasis. Left, the mechanistic sequence from gut dysbiosis to cutaneous inflammation, via barrier damage, LPS translocation, altered microbial metabolites, and immune reprogramming. Right, interventions mapped to their primary targets: probiotics/synbiotics and FMT (investigational) target the microbiota; diet/prebiotics support SCFA production; and immune-level agents include ustekinumab (IL-12/23 p40), risankizumab/guselkumab (IL-23 p19, IBD-efficacious with relative gut-IL-17 sparing), secukinumab/ixekizumab (IL-17A, gut monitoring required), brodalumab (IL-17RA, broad blockade with highest gut-safety concern), and deucravacitinib (oral small-molecule TYK2 inhibitor, not a biologic; signals downstream of IL-23, IL-12, and type I interferons; JAK1/2/3-sparing). The dashed box indicates an unproven probiotic/diet/FMT adjunct for patients on IL-17-pathway inhibitors. Solid arrows, mechanistic sequence; dashed arrows, intervention target. FMT, fecal microbiota transplantation; SCFA, short-chain fatty acid; TYK2, tyrosine kinase 2.

### 5.1 Probiotics and synbiotics

Probiotics are the most extensively studied microbiome-directed intervention. In a 12-week double-blind randomized trial of 44 patients, adding a probiotic supplement to topical corticosteroids produced a substantially larger reduction in Psoriasis Area and Severity Index (PASI) than placebo (46% vs. 15%, p = 0.002), together with better quality-of-life and treatment-satisfaction scores and no reported adverse events (10). The evidence base nonetheless remains limited to small, short-term studies. In psoriatic arthritis, a double-blind pilot trial of a multi-strain probiotic capsule reported immunomodulatory effects (45); whether these findings extend to skin psoriasis remains to be tested. Systematic reviews take a cautiously optimistic view but emphasize the shortage of adequately powered trials (46). Animal data suggest that specific strains, such as Bifidobacterium longum, can preserve mucosal protection and modulate propionate without compromising the efficacy of psoriasis therapy (47).

### 5.2 Fecal microbiota transplantation

FMT aims to restore a healthy microbial community and is considered a promising but poorly evidenced intervention. Acceptance of FMT is relatively high in some populations, although the survey evidence derives mainly from patients with inflammatory bowel disease rather than psoriasis (11). A case report in a patient with ulcerative colitis described resolution of recurrent fungal infection after washed microbiota transplantation, with cutaneous manifestations also improving (48). To date, no randomized trial of FMT in psoriasis has been reported; the intervention should therefore be regarded as experimental in this disease. Donor screening, transplantation protocols, and efficacy endpoints need to be standardized before such trials can be designed (46, 49).

### 5.3 Diet and drug–microbiota interactions

Diet shapes skin inflammation partly through the microbiota: a diet rich in processed food aggravates cutaneous inflammation (50), and nutritional interventions have attracted attention in psoriasis management (51, 52). Drug–microbiota interactions are also relevant. Antibiotic use is associated with the persistence of biologic therapy (53), and biologics themselves alter the microbiota (15, 17). This effect is not uniform across drug classes: IL-17A inhibitors change the gut and scalp microbiota (15, 16), whereas IL-23 inhibitors spare gut-protective IL-17 and might leave a different, potentially safer microbial signature (17). Because SCFA-producing taxa are depleted in psoriasis and SCFAs support both gut-barrier integrity and Treg expansion (30, 31), restoring these taxa through probiotics, diet, or FMT has been proposed as a hypothetical adjunct for patients on IL-17 inhibitors, but this strategy is unproven and has not been tested for reducing gastrointestinal risk. Pharmacomicrobiomics is emerging as a direction for personalized treatment (54).

### 5.4 Evidence quality and clinical feasibility

Applying a GRADE-like framework, the certainty of evidence is moderate for probiotics [one randomized trial showing PASI improvement (10), supported by a psoriatic arthritis pilot trial (45) and preclinical data (47)], low for diet-based approaches (50, 51), and very low for FMT, which rests on survey data and case reports rather than controlled trials (11, 48). Individual responses are likely to vary with baseline microbiota composition, yet no trial has stratified patients accordingly. Safety has received little attention in this literature; FMT in particular carries risks of pathogen transmission and adverse events that must be addressed by donor screening and registry-based follow-up. Future trials should prespecify the target population (e.g., by baseline dysbiosis), the comparator, clinically meaningful endpoints such as PASI 75, and sample sizes powered for effect-size estimation rather than statistical significance alone (46, 49).

## 6 Open questions and outlook

### 6.1 Limited causal evidence

Most human studies are cross-sectional or involve small samples, and cannot distinguish whether microbial changes are a cause, a correlate, or a consequence of psoriasis (6, 23). Mendelian randomization analyses have begun to use genetic instruments to assess causal relationships between the gut microbiota and psoriasis or psoriatic arthritis (55), but their power is constrained by the low heritability of microbial traits and by weak instruments. In addition, most microbiome genome-wide association studies remain too small to detect modest allelic effects, so the instruments explain only a tiny fraction of phenotypic variance; this limits the precision of causal estimates and raises the risk of false-negative results. Prospective cohorts, longitudinal sampling, and interventional designs, including randomized trials of FMT, will be required to establish causality.

### 6.2 Methodological heterogeneity and the need for standardization

Discrepancies between matched-pair and conventional case-control studies point to the influence of methodological heterogeneity (12, 18). Methodological choices have direct consequences: 16S amplicon sequencing reaches genus-level resolution at best and is sensitive to the primer region (e.g., V1–V3 vs. V3–V4) and the reference database used, whereas metagenomic sequencing offers species-level resolution but is more demanding in cost and analytic pipeline. For example, primer selection can materially shift apparent community composition: 16S surveys using V1–V3 versus V3–V4 regions can yield different Firmicutes/Bacteroidetes ratios in the same fecal samples (56), and such technical variation can mimic or mask between-group differences attributed to psoriasis. Database choice and bioinformatic parameters add a further layer of variability, which argues for reporting sequencing and analytic details transparently. Standardizing sample collection and DNA extraction, sequencing strategy, bioinformatic pipelines, confounder control (BMI, diet, medication), and effect-size reporting would reduce avoidable heterogeneity (12, 18).

### 6.3 Future directions

Future work should focus on: (1) multi-omics integration of microbiota, metabolites, immunity, and clinical outcome to build causal networks (57, 58); (2) validation of metabolite-driven mechanisms in humans, particularly the I3S–AhR and SCFA–HCAR2 pathways (9, 30, 59); (3) well-designed clinical trials of FMT and strain-specific interventions (11, 46); and (4) longitudinal cohorts to determine the temporal order and dose-response relationships between the microbiota and disease (55).
